# Optimized fractionated radiotherapy with anti-PD-L1 and anti-TIGIT: a promising new combination

**DOI:** 10.1186/s40425-019-0634-9

**Published:** 2019-06-25

**Authors:** Mathieu Grapin, Corentin Richard, Emeric Limagne, Romain Boidot, Véronique Morgand, Aurélie Bertaut, Valentin Derangere, Pierre-Antoine Laurent, Marion Thibaudin, Jean David Fumet, Gilles Crehange, François Ghiringhelli, Céline Mirjolet

**Affiliations:** 1 Department of Radiation Oncology, Unicancer - Georges-Francois Leclerc Cancer Center, 1 rue Professeur Marion 77 980, 21079 Dijon Cedex, BP France; 2 Cancer Biology Research Platform, Unicancer - Georges-Francois Leclerc Cancer Center, Dijon, France; 3 INSERM UMR 1231, Dijon, France; 4 Methodology, data-management and biostatistics unit, Unicancer - Georges-Francois Leclerc Cancer Center , Dijon, France; 5 Department of Medical Oncology, Unicancer - Georges-Francois Leclerc Cancer Center, Dijon, France

**Keywords:** Radiotherapy fractionation, Immunotherapy, Immune response, Colorectal cancer

## Abstract

**Purpose/objective:**

Radiotherapy (RT) induces an immunogenic antitumor response, but also some immunosuppressive barriers. It remains unclear how different fractionation protocols can modulate the immune microenvironment. Clinical studies are ongoing to evaluate immune checkpoint inhibitors (ICI) in association with RT. However, only few trials aim to optimize the RT fractionation to improve efficacy of these associations. Here we sought to characterize the effect of different fractionation protocols on immune response with a view to associating them with ICI.

**Materials/methods:**

Mice bearing subcutaneous CT26 colon tumors were irradiated using a SARRP device according to different radiation schemes with a same biologically effective dose. Mice were monitored for tumor growth. The radiation immune response (lymphoid, myeloid cells, lymphoid cytokines and immune checkpoint targets) was monitored by flow cytometry at different timepoints after treatment and by RNA sequencing analysis (RNAseq)**.** The same radiation protocols were performed with and without inhibitors of immune checkpoints modulated by RT.

**Results:**

In the absence of ICI, we showed that 18x2Gy and 3x8Gy induced the longest tumor growth delay compared to 1×16.4Gy. While 3x8Gy and 1×16.4Gy induced a lymphoid response (CD8^+^ T-cells, Regulators T-cells), 18x2Gy induced a myeloid response (myeloid-derived suppressor cells, tumor-associated macrophages 2). The secretion of granzyme B by CD8^+^ T cells was increased to a greater extent with 3x8Gy. The expression of PD-L1 by tumor cells was moderately increased by RT, but most durably with 18x2Gy. T cell immunoreceptor with Ig and ITIM domains (TIGIT) expression by CD8^+^ T-cells was increased with 3x8Gy, but decreased with 18x2Gy. These results were also observed with RNAseq. RT was dramatically more effective with 3x8Gy compared to all the other treatments schemes when associated with anti-TIGIT and anti-PD-L1 (9/10 mice in complete response). The association of anti-PD-L1 and RT was also effective in the 18x2Gy group (8/12 mice in complete response).

**Conclusion:**

Each fractionation scheme induced different lymphoid and myeloid responses as well as various modulations of PD-L1 and TIGIT expression. Furthermore, 3x8Gy was the most effective protocol when associated with anti-PD-L1 and anti-TIGIT. This is the first study combining RT and anti-TIGIT with promising results; further studies are warranted.

**Electronic supplementary material:**

The online version of this article (10.1186/s40425-019-0634-9) contains supplementary material, which is available to authorized users.

## Introduction

Radiotherapy (RT) counts among the major anti-cancer treatments, along with surgery, chemotherapy with targeted therapy and immunotherapy. Around 60% of patients with cancer receive RT with curative or palliative intent [1]. Ionizing radiation from RT induces damage to deoxyribonucleic acid (DNA), such as double-strand breaks, which are responsible for mitotic death, as represented by the linear quadratic (LQ) model [2]. The LQ model, developed from in vitro cell survival to RT, predicts the radio-sensitivity to dose per fraction (fractionation) according to cell type, thus defining the biologically effective dose (BED). For years, the biological effect of RT was portrayed only as the DNA effect, modelized in vitro by the LQ model. It is now clear that RT can also modulate the tumoral microenvironment, notably the immune system [3–5]. RT contributes to local and systemic tumor control (the abscopal effect) [6]. The systemic effect of RT is now known to be mediated by the immune system with its capacity to increase CD8^+^ cytotoxic T cells [3]. The recent development of immunotherapy, such as immune checkpoint inhibitors (ICI), makes it possible, when combined with RT, to enhance immune antitumor effects [7–9]. ICI block the ligand/receptor-mediated inhibition of T cells that usually follows T cell activation. There is a strong preclinical rationale underpinning the association of ICI and RT. Administration of an anti-PD-L1 enhances the efficacy of RT through a cytotoxic T cell dependent mechanism [10] even with fractionated RT, which induces upregulation of programmed death-ligand 1 (PD-L1) [11]. Many clinical studies of ICI combined with RT are ongoing in solid cancers. However, the optimal radiation pattern (total dose and fractionation schedule) to stimulate local and abscopal antitumor immune response remains still unclear. In several studies, the different monitored fractionation protocols did not have the same BED. For example, Schaue et al. compared 1x15Gy, 2×7.5Gy, 3x5Gy and 5x3Gy [12] while Vanpouille-Box et al. compared 1x30Gy with 3x8Gy [13]. Vanpouille-Box et al. established a relation between the fractionation dose and antitumor immune response through the DNA exonuclease Trex1, which is induced by radiation doses above 12–18 Gy in different cancer cells, and attenuates their immunogenicity by degrading DNA that accumulates in the cytosol upon radiation. RT could also increase immunosuppressive cells [14–16], by different mechanisms according to the radiation scheme. Radiation regimens have to be optimized to improve antitumor immune response for successful combination with other treatments, including ICI. We also observed in rectal cancer that the dose fractionation differently influenced CD8+/Regulatory T-cells (Treg), a tumor-infiltrating-lymphocytes (TILs) ratio, which was predictive of prognosis [17]. Our goal was to study how the dose per fraction can modulate the immune system, in order to associate specifically an ICI in the setting of subcutaneous transplantable mouse cancer.

## Material and methods

### Cell culture and animals

CT26 American Type Culture Collection (ATCC) murine colon cancer cells (USA) were cultured in RPMI 1640 (Dutscher, France) + 10% fetal calf serum (Dutscher, France) (37 °C, 5% carbon dioxide and 95% humidity). B16-F10 murine melanoma cancer cells (USA) were cultured in DMEM (Dutscher, France) + L-Glutamine + red phenol + glucose (4.5 g/l) + 10% fetal calf serum (Dutscher, France) (37 °C, 5% carbon dioxide and 95% humidity).

The day before mice were injected with cancer cells. These cells were contacted with trypsin and diluted to ½. The unit injection included 5 × 10^5^ CT26 cells in 100 μl of NaCl, or 1 × 10^6^ B16-F10 cells 100 μl of NaCl, in performed subcutaneously on the right flank of immunocompetent BALB/c female and C57BL female mice and 8-week immunosuppressed athymic BALB/c nude mice (Charles River Laboratories, Saint-Germain-des-Monts, France). During the entire duration of the experiment, mice were housed in our approved animal facility (Centre Georges-François Leclerc, Dijon, FRANCE). The mice were sacrificed by cervical dislocation after Isoflurane 2.5% anesthesia as soon as a limit point was reached (Tumoral Volume (TV) ≥1500 mm3, pain, significant necrosis).

Before experimentation, the small animal ethics committee and the Ministry of Higher Education and Research validated the project.

### Treatments

Ten days after injection of cancer cells, randomization was performed to distribute the mice to the different treatment groups, to obtain an equivalent average TV in each treatment group (about 150 mm^3^). The BED was calculated using the LQ model (BED = D (1 + d / [α/β]), with D = total dose, d = dose per fraction, α/β = 10) [18]. Retaining an α/β = 10 ratio for tumor tissue, we developed 3 RT schemes with a BED = 43.2Gy: 18 fractions of 2Gy (18x2Gy), 3 fractions of 8Gy (3x8Gy), 1 fraction of 16.4Gy (1×16.4Gy). Before and during irradiation, each mouse was anesthetized with 2.5% Isoflurane mixed with oxygen (MINERVE system, France).

Radiotherapy was delivered by a small animal irradiator (SARRP, Xstrahl, UK), with 225 kV energy X-ray photons, and a dose rate of 3.1 Gy/min [19]. For each RT session, an anterior field and a posterior field were used to irradiate the tumor in a targeted way with a homogeneous dose.

Intra-peritoneal injections of Immunoglobulin G (IgG) (BioXcel, USA) and anti-PD-L1 (BioXcel, USA) were performed 3 times per week for 3 weeks, starting from the 1st day of RT, at a dose of 10 mg/kg per injection. Intra-peritoneal injections of anti-T cell immunoreceptor with Ig and ITIM domains (TIGIT) (BioXcel, USA) were performed twice a week for 3 weeks, starting from the 1st day of RT, at a dose of 10 mg/kg per injection. The injected volume per mouse per injection was 100 μL. For B16-F10 only 3x8Gy was evaluated, in association with IgG, anti-PD-L1, anti-TIGIT, and anti-TIGIT + anti-PD-L1.

### Treatment effects

To evaluate the effectiveness of treatment, tumor growth was evaluated by the growth retardation parameter (time to reach a volume of 1500 mm^3^) and survival. The TV was recorded 3 times a week using calipers and calculated according to the following formula: TV = width x width x length × 0.5. Treatment efficacy was also assessed by the number of mice in CR / total number of mice treated in the same group. Survival time was counted from the day of randomization to death (TV ≥1500 mm3, pain, significant necrosis). Each group included from 6 to 12 mice; the numbers were calculated taking into account inter-mouse variability.

### Flow cytometry

The modulation of the immune system by the different fractionation schemes was evaluated by flow cytometry (FCM), after labeling the cells with antibodies specific for different types of immune cells (Table 4) at different treatment time points (control group, 7 and 14 days after the first RT session for 1×16,4Gy, 3x8Gy and 18x2Gy and 7 days after the last session (30 days after the first RT session) for 18x2Gy) (Fig. 2a).

The different kinetic points of the analysis were intended to compare each RT group with the control group or between each RT schedule in two different ways: chronologically or in relation to the dose delivered, to take into account both the spread, and the total dose delivered.

After dissection, tumors were mechanically and enzymatically dissociated using a mouse tumor dissociation kit according to manufacturer’s recommendation (Miltenyi Biotech). To analyse myeloid cell infiltration, the tumor cell suspension (10^6^ cells) was stained in Flow cytometry Staining Buffer (FSB, eBioscience) with specific antibodies according to manufacturer’s recommendation (antibody details are presented in Additional file 1: Table S1) during 15 min at RT in dark, washed twice in FSB and analyzed by flow cytometry. To analyze lymphoid cell infiltration, the tumor cell suspension was stained with the Foxp3 staining buffer set according to manufacturer’s recommendation (Miltenyi Biotech) (antibody details are presented in Additional file 1: Table S1). For lymphoid and myeloid cell infiltration assay, viability dye eFluor 780 was used to identify live cells. Flow cytometry acquisition was performed on Cytoflex 13C cytometer (Beckman Coulter). CytExpert (Beckman Coulter) was used for analysis. For lymphoid and myeloid cells identification see gating strategy presented in Additional file 2: Figures S1 and S2.

To study cytokine function of the lymphoid infiltrate the tumor cell suspension was cultured on 96-well plates with complete RPMI medium (Dutscher) overnight at 37 °C. During the last 4 h of culture PMA (phorbol 12-myristate 13-acetate; 20 ng/ml; Sigma-Aldrich), ionomycin (1 μg/ml; Sigma-Aldrich), and brefeldin A (2 μl/ml; eBioscience) were added. After staining of surface markers (antibody details in Additional file 1**:** Table S1), cells were fixed and permeabilized with Foxp3 staining buffer set according to the manufacturer’s instructions (Miltenyi Biotech), then intracellular proteins were stained (antibody details in Additional file 1**:** Table S2). Viability dye eFluor 780 was used to identify live cells. For Flow cytometry acquisition was performed on Cytoflex 13C cytometer (Beckman Coulter). CytExpert (Beckman Coulter) was used for analysis. For lymphoid functionality see gating strategy presented in Additional file 2: Figure S3.

### RNA extraction, RNA sequencing (RNAseq) and gene set enrichment analysis

For each tumor sample, RNA extraction was performed with Trizol reagent (Invitrogen) after tissue dissociation using Minilys tissue homogenizer (Bertin, Ozyme). Total RNA was extracted from tumor using the Trizol method. Libraries were prepared from 1 μg of total RNA with the TruSeq Stranded Total RNA using Ribo-Zero (Illumina) following the manufacturer’s instructions. Once qualified, single-end libraries were sequenced using 1 × 76 bp output on a NextSeq 500 device (Illumina).

Paired-end transcriptome reads were pseudoaligned to the UCSC mm 10 reference genome and quantification of gene expressions as TPM (Transcript per Million) value were performed with the Kallisto algorithm [20]. The program was run with default options. Differential analysis was performed with DESeq2 R package [21] using log fold change shrinkage. A gene was considered significantly differentially expressed when the corresponding s-value < 0.005.

A gene set enrichment analysis was performed using the Cytoscape plug-in ClueGO [22] and the databases GO and KEGG 2018. The app was run using default parameters.

### Statistical analysis

The results were expressed as mean ± standard error of the mean (SEM). All figures were produced using GraphPad Prism software (Graphpad Software, USA). Differences in survival were analyzed by the Logrank test. Comparisons between groups were performed using a non-parametric Mann-Whitney test. Statistical analyses were performed using SAS version 9.4 (SAS Institute Inc., Cary, NC, USA). A *p* value less than 0.05 was considered to be statistically significant.

## Results

### Comparison of different RT schemes with or without immune system

**Fig. 1:**
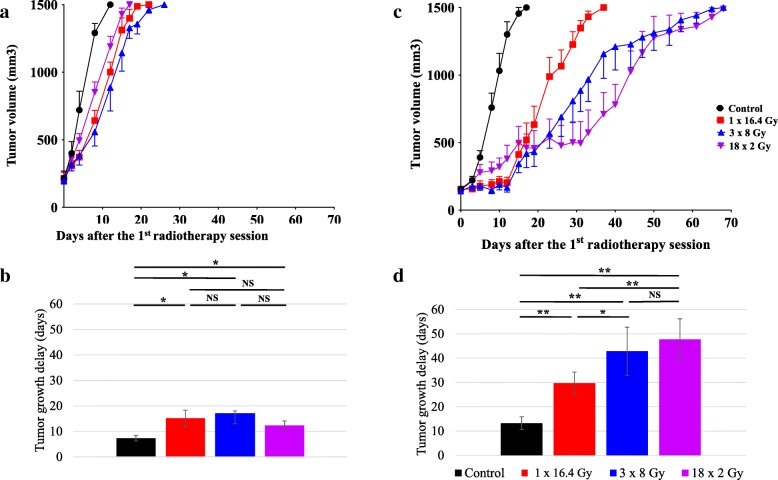
Effect of fractionation of RT on CT26 tumors grafted onto immunodepressed (**a**, **b**) or immunocompetent (c, d) mice. Growth of irradiated tumors in immunodeficient BALB/C nude mice (**a**) (*n* = 6 mice per group) or immunocompetent BALB/C mice (**c**) (*n* = 10–12 mice per group) treated with: 0Gy (black), 1×16.4Gy (red), 3x8Gy (blue), 18x2Gy (purple). The averages are expressed ± SEM The average time for the tumor volume to reach 1500 mm^3^ in each group is shown for immunodepressed mice (**b**) or immunocompetent mice (**d**). Not significant (NS); *p < 0.05; **p < 0.01. Non-parametric Mann-Whitney test was used

In order to evaluate the role of immune system on different RT schedules we monitored the growth of tumors implanted on the flank of immunodeficient and immunocompetent mice. We select 3 schedules with similar BED (18x2Gy, 3x8Gy, 1×16.4Gy). In BALB/c nude mice, we showed that the different RT schedules induced similar antitumor effect (Fig. 1 a). There was no significant difference in the time to reach a tumor volume of 1500 mm^3^ (Fig. 1 b). We performed the same experiment on BALB/c immunocompetent mice. In untreated mice, tumor grows faster than in BALB/c nude mice. In these mice, we observed that different RT regimens induced different tumor control. As shown in Fig. 1 c, the effectiveness of the 18x2Gy scheme was delayed and appeared after the end of treatment, which was spread over 24 days. Compared to the 1×16.4Gy scheme, the time for the TV to reach 1500 mm^3^ was longest with the 18x2Gy (*p* = 0.001) and 3x8Gy schemes (*p* = 0.02) (Fig. 1 d). Nevertheless, there was no significant difference between these latter two schemes (*p* = 0.20).

### 3x8Gy and 1 × 16.4 Gy rapidly increased lymphoid cells

**Fig. 2:**
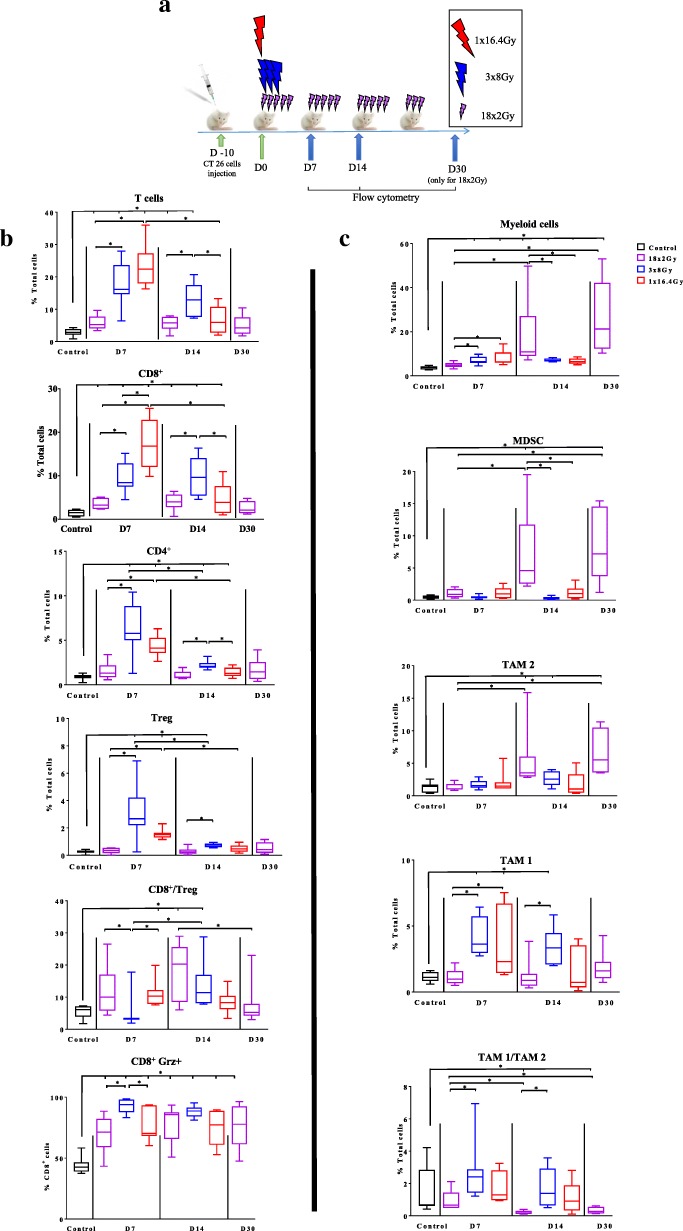
Immunomonitoring of lymphoid cells and myeloid cells after radiotherapy. Ten days after the injection of CT26 colon murine cancer, mice were assigned in 4 groups: control (at day 7), 1×16.4Gy (red), 3x8Gy (blue), 18x2Gy (purple) (**a**). Seven, 14 and 30 days after the beginning of RT, flow cytometry monitoring (FCM) was performed on dissociated tumors. Lymphoid panel analysis (**b**) including: T-cells, CD8^+^ T cells, CD4^+^ T cells, Treg T cells, CD8^+^ T cells/CD4^+^ T cells ratio, CD8^+^ granzyme^+^ (grz). Myeloid panel analysis (**c**) including: myeloid cells, myeloid-derived suppressor cells (MDSC), tumor-associated macrophages (TAM) 2, TAM 1, TAM1/TAM2 ratio. All data are shown with box and whiskers with min to max values obtained from 8 independent samples per point (duplicate, *n* = 8 per condition). *p < 0.05. Non-parametric Mann-Whitney test was used

To understand the mechanisms leading to the antitumor effect after the different RT schemes, we performed immunomonitoring using FCM at different time points for each scheme, as described above (Fig. 2 a). Control condition was evaluated at Day7. No significant differences in T cell tumor infiltration was observed in control tumor at Day 0, Day 7 and Day14 (data not shown). The variations in tumor infiltrated lymphoid cells are shown in Fig. 2 b**.** All immunomonitoring data were expressed in % of total cells including cancer cells and immune cells in the tumor. Seven days after the first RT fraction, the 1×16.4Gy and 3x8Gy schemes induced significant accumulation of total T-cells (24.0% ± 2.5 and 17.6% ± 2.4% respectively) compared to the 18x2Gy group (5.9% ± 0.8%) and the control group (2.9% ± 0.4%) (*p* < 0.001). Then T-cells decreased progressively at day 14. The maximal lymphoid shrinkage was observed with the 1×16.4Gy schedule. With the 3x8Gy scheme, the lymphoid population accumulation was maintained with a modest decrease.

The variations observed in total T lymphocytes were similar to those observed in CD8^+^ T cells. Indeed, the proportion of CD8^+^ T cells to the total cells 7 days after the first RT session was 17.0% ± 2.2% in the 1 × 16.4Gy group (*p* = 0.002), 9.6% ± 1.2% for 3x8Gy group (*p* < 0.001), 3.6% ± 0.4% in the 18x2Gy group (p < 0.001); versus 1.4% ± 0.3% in the control group. At the next kinetic time point, the increase in CD8^+^ T cells remained significant with the 3x8Gy scheme (9.8% ± 1.6%) compared to 1×16.4Gy (4.5% ± 1.3%) (*p* = 0.04); 18x2Gy (3.9% ± 0.7%) (*p* = 0.02) and control groups (p < 0.001).

The proportion of CD4^+^ T cells was significantly increased 7 days after the first RT session in the mono-fractionated and 3x8Gy groups compared to the control group: 4.3% ± 0.5% (p = 0.002) and 6.2% ± 1.0% (*p* = 0.001) respectively; and decreased significantly at day 14. The variations observed on the CD4^+^ T lymphocytes were similar to the Treg findings, which represented a large proportion of CD4^+^ T lymphocytes. There was no significant effect of the 18x2Gy on the proportion of Treg cells.

After 3x8Gy, the CD8^+^/Treg ratio was lowest (4.9 ± 1.8), compared to 1×16.4Gy (11.2 ± 1.6) (*p* = 0.03) and 18x2Gy (121 ± 2.9) (p = 0.03) at day 7. The CD8^+^/Treg ratio at day 14 was non significantly different between the RT schedules, although there was a tendency for the ratio to increase, the more RT was fractionated (*p* = 0.07).

Radiotherapy, whatever the regimen used, significantly increased the proportion of functional CD8^+^ T cells secreting granzyme B compared to the control group. This increase, which appeared from the first week, was maintained until two weeks after the end of the irradiation. The 3x8Gy scheme induced the highest proportion of CD8^+^ T cells secreting granzyme B at day 7 (92.8% ± 2.0%) compared to 18x2Gy (70.3% ± 5.2%) (*p* = 0.005) and 1×16.4Gy (76.6% ± 4.9%) (*p* = 0.04).

### 18x2Gy increased immunosuppressive myeloid cells in a delayed but prolonged manner

The variations in tumor infiltrated myeloid cells are shown in Fig. 2 c **.**The proportion of total myeloid cells increased significantly in the 18x2Gy group from day 14 (17.9% ± 5.3% at day 14 and 26.9% ± 5.7% at day 30) compared to the other radiotherapy groups (p = 0.04) and the control group (2.6% ± 0.8%) (*p* < 0.001). Similar findings were observed for MDSC and TAM2. A significant increase in TAM1 was observed with 3x8Gy at day 7 (4.1% ± 0.5%) (p < 0.001) and day 14 (3.4% ± 0.5%) (p < 0.001) compared to control group.

The TAM1/TAM2 ratio was increased in 3x8Gy group at day 14 (1.8% ± 0.4%) compared to the control group (1.5% ± 0.5%). At day 14 and day 30, the TAM1/TAM2 ratio was significantly lower with 18x2Gy compared to the control group (0.2% ± 0.036% (p < 0.001) and 0.3% ± 0.07% (*p* = 0.003) respectively).

### RNA sequencing analysis

**Fig. 3:**
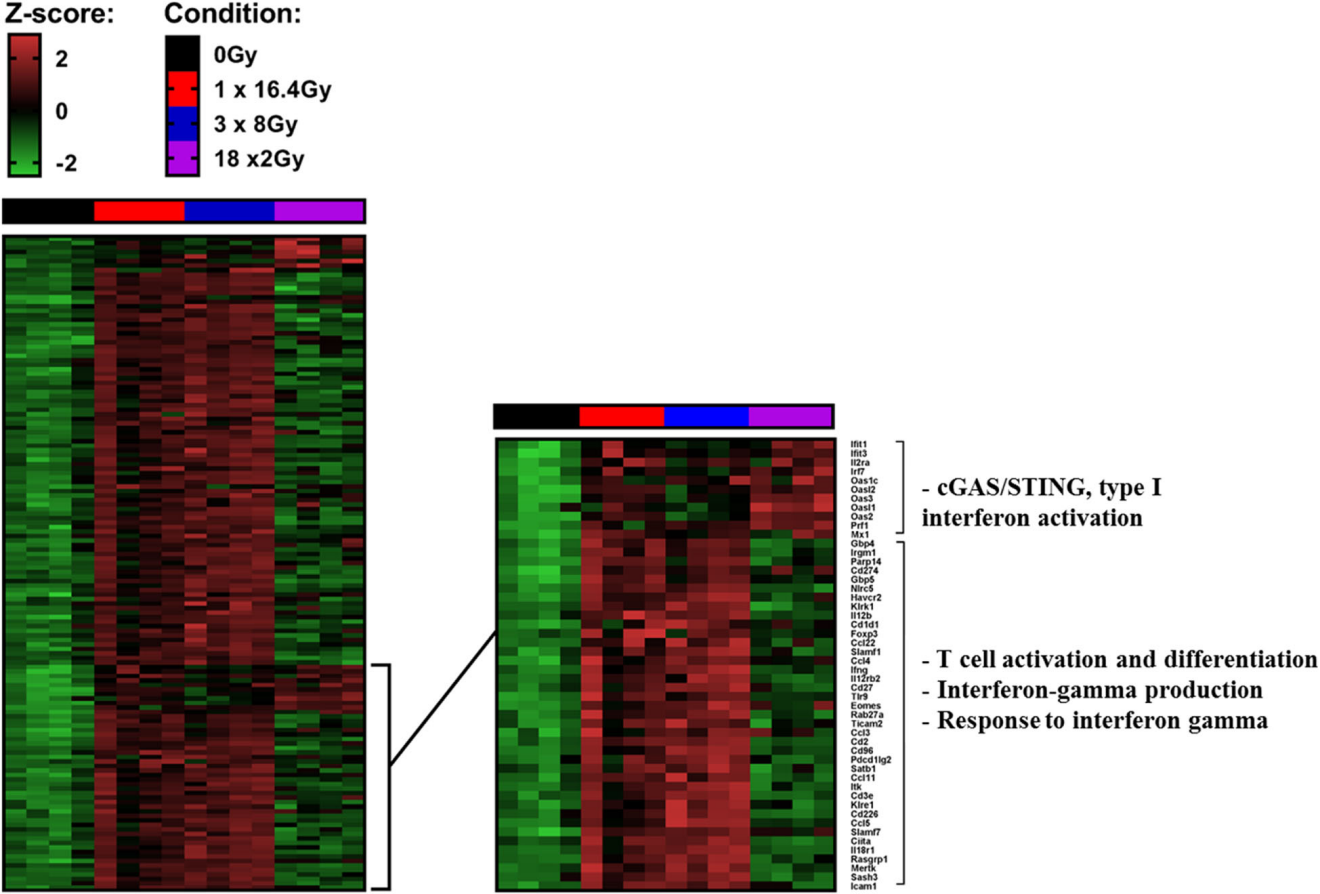
Heatmaps showing differentially expressed genes at day 7 after the end of treatment tumors (CT26 model) between at least one condition and control group. Illustration of gene expression with s-value < 0.005 and absolute shrink lock-fold change threshold of one (Z-score): control (black), 1×16.4Gy (red), 3x8Gy (blue), 18x2Gy (purple). Experimental groups contained 4 mice per condition

Gene transcripts were analyzed in each group 7 days after the beginning of RT and 7 days after the completion of RT in the 18x2Gy group. A large majority of genes are overexpressed with the two hypofractionated schemes compared to 18x2Gy. We notably observed increased expression of genes associated with CD8^+^ T cell activation and differentiation, interferon gamma production and response pathways (Fig. 3). On the contrary, c-GAS STING pathway activation was mostly upregulated in 18x2Gy.

### A specific ICI for each fractionated RT scheme to improve efficacy

**Fig. 4:**
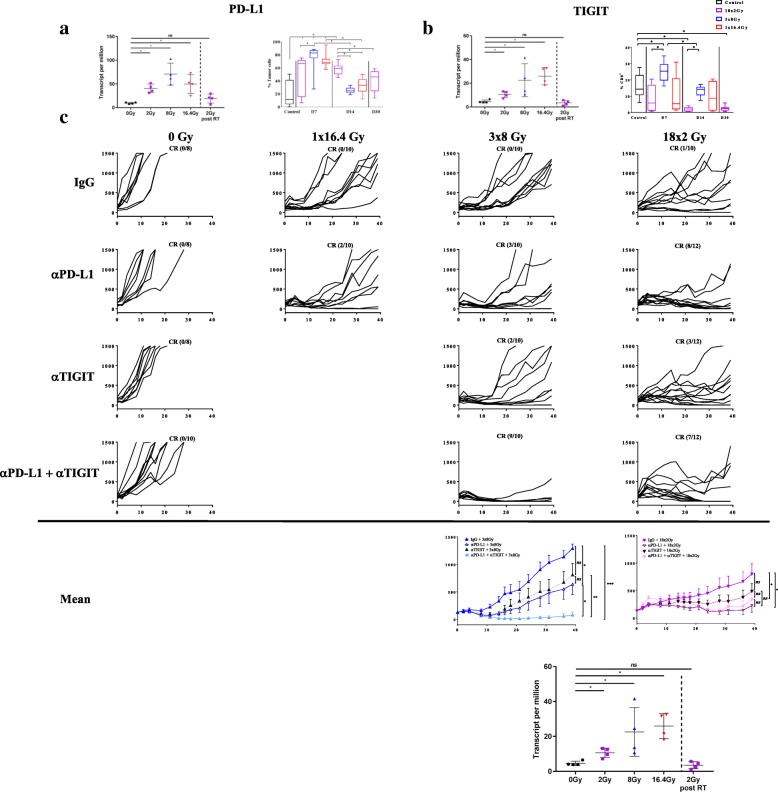
Efficacy evaluation of immunotherapy (anti-PD-L1 and/or anti-TIGIT) and different fractionation schemes of radiotherapy (RT) in CT26 model. Induction of the expression of PD-L1 (cd274 gene) (**a**) or TIGIT (**b**) using RNA sequencing analysis (left) (7 days after the beginning of RT and 7 days after the end of RT for the 18x2Gy scheme) and flow cytometry monitoring (FCM) (right) (7, 14 days after the beginning of RT and 7 days after the end of RT (day 30) for the 18x2Gy scheme): control (black), 1×16.4Gy (red), 3x8Gy (blue), 18x2Gy (purple). Growth of irradiated tumors in mice treated with 0Gy, 1×16.4Gy, 3x8Gy, 18x2Gy with IgG or anti-PD-L1 and/or anti-TIGIT (**c**). Complete response (CR) ratio indicates the number of mice free from the irradiated tumor. Mean ± SEM for 18x2Gy (purple) and 3x8Gy (blue) are shown at the bottom of the Fig. X axes express the number of days since the beginning of RT. Y axes express the tumor volume (mm^3^). Experimental groups contained at least 8 mice per group. Not significant (NS); *p < 0.05; **p < 0.01, ***p < 0.001. Non-parametric Mann-Whitney test was used

As shown in Fig. 4 a with RNA sequencing analysis and then with FCM, RT significantly increased the expression of PD-L1 on tumor cells, whatever the scheme, at day 7 compared to the control group. At day 14, tumoral expression of PD-L1 remained high only with 18x2Gy (58.6% ± 3.1%) compared to the control group (18.7% ± 7.1%) (*p* = 0.004). At day 30, the expression of PD-L1 was non-significantly increased (41.4% ± 5.8%) (*p* = 0.07) although there was a trend in the 18x2Gy group.

Figure 4 b shows that 3x8Gy increased TIGIT expression on CD8^+^ T cells at day 7 (25.3% ± 2.2%) compared to the control group (16.1% ± 2.5%) (*p* = 0.02) and the 18x2Gy group (8.6% ± 2.9) (*p* = 0.009). At day 14 the expression of TIGIT was higher in the 3x8Gy group (13.3% ± 1.2%) compared to the 18x2Gy group (2.0% ± 0.5%) (*p* = 0.002). In the 18x2Gy group the expression of TIGIT decreased progressively at day 7, day 14 and day 30 (2.4% ± 0.6%) compared to the control group (*p* < 0.001).

The anti-PD-L1 and anti-TIGIT did not have any antitumor effect alone **(**Fig. 4 c). The association of anti-PD-L1 with RT increased tumor control compared to IgG with RT, and the anti–tumor response was the most effective with the fractionated groups and especially with 18x2Gy (8/12 CR). Mean tumor volume at day 39 was significantly lower in the 18x2Gy + anti-PD-L1 group (*p* = 0.01) and 18x2Gy + anti-PD-L1 + anti-TIGIT group (*p* = 0.04) compared to the 18x2Gy group.

Anti-TIGIT in association with RT was not significantly effective compared to IgG with RT, whatever the fractionation scheme.

The association of anti-TIGIT, anti-PD-L1 and 3x8Gy (9/10 CR) was the most effective compared to all other groups: 3x8Gy + anti-PD-L1 (3/10 CR), 3x8Gy + anti-TIGIT (2/10 CR). Mean tumor volume at day 39 was lowest in the 3x8Gy + anti-PD-L1 + anti-TIGIT group (*p* < 0.05) compared to all the other 3x8Gy groups. The 18x2Gy group did not benefit from the dual ICI (7/12 CR) compared to 18x2Gy + anti-PD-L1 (8/12 CR).

**Fig. 5:**
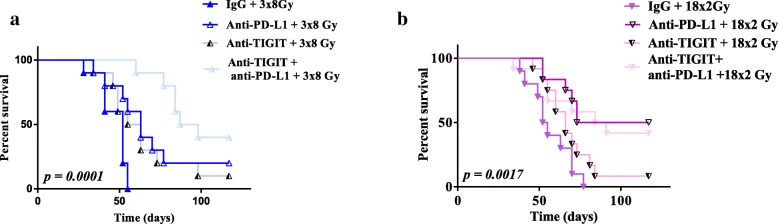
Survival curves after immunotherapy (anti-PD-L1 and/or anti-TIGIT) and fractionated radiotherapy (RT) in CT26 model. Survival curves of mice treated with 3x8Gy (**a**), 18x2Gy (**b**) with IgG or anti-PD-L1 and/or anti-TIGIT. X axes express the number of days since the beginning of RT. Y axes express the percentage survival of mice in each group. Experimental groups contained at least 10 mice per group. Log-rank test was used

On the one hand, anti-TIGIT yielded a significant antitumor effect only when associated with anti-PD-L1 and the 3x8Gy scheme. On the other hand, there was no significant antitumor effect of anti-TIGIT when associated with 18x2Gy, or 18x2Gy + anti-PD-L1 (Fig. 5).

In the B16-F10 model, 3x8Gy tended to be more effective when associated with anti-TIGIT + anti-PD-L1 compared to anti-TIGIT alone, anti-PD-L1 alone, or IgG (*p* = 0.06, *n* = 5 per group) (Additional file 2**:** Figure S4).

## Discussion

Our study aimed to define changes in the tumor immune microenvironment induced by different dose per fraction schemes (with a same BED), and to identify on the one hand, factors leading to immune suppression and resistance to RT, and on the other hand, factors leading to activation of antitumor immunity, with a view to adapting the association of an ICI. Firstly, we validated the dose equivalence between the different fractionation protocols in the absence of an immune system and showed that the two fractionated schemes were more effective. As BALC/c mice and BALC/c nude mice have a common genetic background, differences observed in tumor growth are due to the immune system. The results obtained at this stage with the normo-fractionated scheme are particularly interesting. Indeed, essentially schemes with repeated doses per fraction between 6Gy and 12Gy were considered as pro-immunogenic, whether in pre-clinical studies [12, 23] or in clinical studies [24, 25]. Few studies compared dose fractionation schemes with a same BED, and no studies have evaluated fractionated schemes with more than 9 fractions [15]. Our immuno-monitoring of these different RT schemes was intended to help us understand the underlying mechanisms of the immune response. Without treatment we observed a low Lymphoid T cell infiltration representing 2.87% +/− 1.12 of total tumor cells (mean+/−SD). CD8+ T cell represented 1.42% +/− 0.73 of total tumor cells. These results were comparable to previous studies on CT26 models [26, 27]. The 1×16.4Gy and 3x8Gy hypo-fractionated schemes induced an intense, brief and predominantly lymphoid immune response 7 days after irradiation. The 18x2Gy pattern, on the other hand, induced a predominantly myeloid response 2 weeks after the beginning of the irradiation, which persisted over time. The effect of hypo-fractionation appears to be critical for lymphoid stimulation, while normo-fractionation seems to be deleterious to lymphoid cells, which are radiosensitive. This may be explained by the fact that when the lymphoid cells infiltrate the tumor several days after the first session, the tumor continues to be irradiated; or that circulating lymphocytes in the vascular system near the irradiation field are repeatedly irradiated, resulting in lymphopenia [28, 29]. Recently, a study in a model of LL/2 lung cancer and B16-F10 melanoma, compared the effect of the dose per fraction of a so-called “conventionally fractionated” pattern (9x4Gy) and another hypo-fractionated (2×11.5Gy), both having a spread of 9 days and the same BED, in combination with an anti-programmed cell death-1 (PD-1) [15]. The authors demonstrated the superiority of the hypo-fractionated regimen on tumor control and explained it by the effect of RT on MDSC, which are markedly diminished in this scheme. These results on different tumor models and non-similar RT regimens were similar to ours for the effect on MDSC, but not on tumor control. The dose per fraction of RT acts in a different way on the polarization of macrophages. Thus, in our study, the two hypo-fractionated schemes (1×16.4Gy and 3x8Gy) induced TAM1, which stimulates inflammation and the anti-tumor immune response, while the normo-fractionated scheme induced TAM2, which promotes tumor growth, angiogenesis, and metastasis [30]. Our results were inconsistent with those found in the literature regarding the effect of fractional dose on TAM [31, 32], although these studies were performed using different cell lines. Nevertheless, TAM2 and MDSC express PD-L1 [33], explaining the beneficial effect of the association of an anti-PD-L1 with normo-fractionated RT. The effect of a normo-fractionated scheme (5x2Gy) regimen on the immune system, with or without PD-L1, in a CT26 model, has already been reported (using a total dose lower than ours) [11]. In this study, the authors showed that normo-fractionated RT induces a specific immune response and memory, with a greater clinical anti-tumor effect than our study when RT is associated with anti-PD-L1.

In the present study, we observed a contrasting effect of hypo fractionated RT versus normo-fractionated schedule. While the first one induced CD8+ T cells recruitment and additional checkpoints, the second one induced MDSC and TAM2 accumulation and a prolonged PD-L1 expression induction. Increasing data underline that CD8 T cells accumulation in tumor bed is a good predictive marker of checkpoint efficacy [34]. In addition, the presence of additional checkpoints, other than PD-1, is a marker of advanced CD8 T cells exhaustion. Normo-fractionated RT induced accumulation of MDSC and TAM2. These two cell populations are known to be associated with poor prognosis in many cancer types [35, 36]. Additionally, recent data also suggest an association between the presence of these cells and resistance to checkpoint inhibitors [37, 38]. The myeloid biomarkers have been less investigated and sparse data are available in the literature. Further work is required to determine if MDSC or TAM2 elimination could reverse resistance to immunotherapy or combination of normo-fractionated RT plus immunotherapy.

Initially, the utility of associating immunotherapy with RT was to amplify the abscopal effect, which was described in literature after hypo-fractionated (6-12Gy per fraction) and repeated RT [12, 13, 23, 39–42]. Normo-fractionated RT (2Gy per fraction) might have an immunosuppressive action [29, 43]. But it is unclear, some clinical studies have shown an anti-tumor immunomodulation effect of normo-fractionated RT, especially when associated with ICI [44, 45]. In many of the studies comparing RT fractionation schemes, the BED (i.e. the cytotoxic effect) was not the same. Thus, several teams have studied the effect of RT on immune activation, most often using a high dose per fraction. In this present study, we highlight the fact that it can also be useful to associated normo-fractionated RT with ICI. However, it seems essential to develop specific biomarkers that describe which targets will be induced by this type of RT schedule. The inferiority of the 1 × 16.4 Gy scheme on tumor control can be explained by the findings of Vanpouille-Box et al. Indeed, these authors showed in a pre-clinical model that doses per fraction greater than 12 Gy induce accumulation in the cytoplasm of an exonuclease called Trex1. Similar results were observed using RNAseq method in the present study (data not shown). Thus, the cytosolic DNA that accumulates in the cytosol during irradiation is degraded. However, when this DNA is present, it stimulates the secretion of interferon β via the pathway stimulator of interferon genes (STING), allowing the recruitment and activation of dendritic cells. Thus, the concentration of cytosolic DNA gradually increases up to a dose of 12 Gy per fraction and then collapses [13]. Vanpouille-Box et al. also suggested in their article an interesting ex-vivo test that may analyze the effect of several types of RT schedules on PDX models produced from patient’s tumors. From analysis of gene expression induced by the cGAS/STING pathway, the authors would like to develop a new factor to describe the RT fractionation scheme that will induce the best immune response, to associate it with immunotherapy. We could suggest a complementary evaluation to this method, namely the analysis of radio-induced immune ICI target expression. As we highlighted in the present study, gene expression induction of these targets, analyzed by RNAseq, correlated with expression analyzed by FCM immuno-monitoring and with related immunotherapy efficacy.

Most patients (≈ 60–80%) will not respond to current ICI such as anti-PD-L1 or anti-PD-1 alone [46, 47] in metastatic solid cancers, for which ICI have shown a clinical benefit (such as melanoma, lung cancer). We showed that CT26 or B16-F10 cancer cells have a poor response to ICI without RT. While the 18x2Gy scheme was most effective with anti-PD-L1 (8/12 CR), the 3x8Gy scheme was the most effective when associated with anti-TIGIT and anti-PD-L1 (9/10). Based on our FCM analyses and to explain these results, we observed firstly that 18x2Gy induced the expression of PD-L1 in a sustainable manner, but significantly decreased the expression of TIGIT. Conversely, the 3x8Gy scheme significantly increased the expression of PD-L1 and TIGIT. TIGIT is a co-inhibitory receptor which can be expressed by CD8+ T cells, natural killer cells, Treg cells and T follicular helper cells [48, 49]. The TIGIT ligands, CD155 and CD112 can be expressed by different cell types, including antigen-presenting cells and tumor cells [50, 51]. TIGIT is associated with CD8+ T cells exhaustion [52, 53]. Johnston et al. studied anti-TIGIT alone or in combination with anti-PD-L1 in a CT26 tumor model [53]. They observed that the majority of mice receiving the combo of ICI were in CR, unlike our results. However, according to our findings, there was no significant effect of anti-TIGIT alone or anti-PD-L1 alone. This is the first study to evaluate the benefit of an anti-TIGIT combined with an optimized RT. We showed promising results of the combination anti-TIGIT + anti-PD-L1 + 3x8Gy, which could be evaluated in a clinical study. We suggest that each fractionation (normo-fractionated or hypo-fractionated) scheme may specifically induce an immune checkpoint (PD-L1 and/or TIGIT) and need an appropriate ICI (respectively anti-PD-L1 or anti-TIGIT).

## Conclusion

Each fractionation scheme induced different lymphoid and myeloid responses, as well as various degrees of modulation of PD-L1 and TIGIT expression. Furthermore, 3x8Gy was the most effective protocol when associated with anti-PD-L1 and anti-TIGIT. On the contrary, the 18x2Gy scheme associated with anti-PD-L1 was not more effective when associated with anti-TIGIT.

This is the first study highlighting the relevance of optimizing RT fractionation schemes for association with ICI, and combining RT and anti-TIGIT with promising results; further studies are warranted.

## Additional files

Additional file 1: **Table S1.** List of antibodies used for identification of myeloid and lymphoid cell and for the study of lymphoid function. (XLSX 13 kb)

Additional file 2: **Figure S1.** Gating strategy for lymphoid cells identification and quantification in tumor tissue. **Figure S2.** Gating strategy for myeloid and tumor cells identification, quantification and phenotype (PD-L1 expression) in tumor tissue. **Figure S3.** Gating strategy for lymphoid cells functionality quantification in tumor tissue. **Figure S4.** Efficacy evaluation of immunotherapy (anti-PD-L1 and/or anti-TIGIT) and RT (3x8Gy) in B16-F10 model. Growth of irradiated tumors in mice treated with IgG + 0Gy (black), IgG + 3x8Gy (red), anti-TIGIT + anti-PD-L1 (blue) and with anti-TIGIT + anti-PD-L1 + 3x8Gy (purple). Mean ± SEM. X axes express the number of days since the beginning of RT. Y axes express the tumor volume (mm^3^). Experimental groups contained at 5 mice per group. Non-parametric Mann-Whitney test was used. **Figure S5.** Immunomonitoring of Treg and CD8+ T cells and their KI67 and PD-1 status after radiotherapy. Ten days after the injection of CT26 colon murine cancer, mice were assigned in 4 groups: control (at day 7), 1 × 16.4Gy (red), 3x8Gy (blue), 18x2Gy (purple) (a). Seven, 14 and 30 days after the beginning of RT, flow cytometry monitoring (FCM) was performed on dissociated tumors. CD8^+^ T cells (a) and Treg T cells (b) were analyzed according to their status for KI67 and PD-1 labelling. All data are shown with box and whiskers with min to max values obtained from 8 independent samples per point (duplicate, *n* = 8 per condition). In the second part of a) and b), representative cytometry analysis was highlighted for each condition at day 7 and day 14 after treatment. *p < 0.05. Non-parametric Mann-Whitney test was used. (ZIP 3298 kb)

## Data Availability

All data generated or analyzed during this study are included in this published article and its supplementary information files.

## References

[1] Orth M, Lauber K, Niyazi M, Friedl AA, Li M, Maihöfer C (2014). Current concepts in clinical radiation oncology. Radiat Environ Biophys.

[2] Fowler JF (1989). The linear-quadratic formula and progress in fractionated radiotherapy. Br J Radiol.

[3] Formenti SC, Demaria S (2009). Systemic effects of local radiotherapy. Lancet Oncol.

[4] Golden EB, Apetoh L (2015). Radiotherapy and immunogenic cell death. Semin Radiat Oncol.

[5] Shahabi V, Postow MA, Tuck D, Wolchok JD (2015). Immune-priming of the tumor microenvironment by radiotherapy: rationale for combination with immunotherapy to improve anticancer efficacy. Am J Clin Oncol.

[6] Demaria S, Ng B, Devitt ML, Babb JS, Kawashima N, Liebes L (2004). Ionizing radiation inhibition of distant untreated tumors (abscopal effect) is immune mediated. Int J Radiat Oncol Biol Phys.

[7] Apetoh L, Ladoire S, Coukos G, Ghiringhelli F (2015). Combining immunotherapy and anticancer agents: the right path to achieve cancer cure?. Ann Oncol Off J Eur Soc Med Oncol.

[8] Tang C, Wang X, Soh H, Seyedin S, Cortez MA, Krishnan S (2014). Combining radiation and immunotherapy: a new systemic therapy for solid tumors?. Cancer Immunol Res.

[9] Ngwa W, Irabor OC, Schoenfeld JD, Hesser J, Demaria S, Formenti SC (2018). Using immunotherapy to boost the abscopal effect. Nat Rev Cancer.

[10] Deng L, Liang H, Burnette B, Beckett M, Darga T, Weichselbaum RR (2014). Irradiation and anti–PD-L1 treatment synergistically promote antitumor immunity in mice. J Clin Invest.

[11] Dovedi SJ, Adlard AL, Lipowska-Bhalla G, McKenna C, Jones S, Cheadle EJ (2014). Acquired resistance to fractionated radiotherapy can be overcome by concurrent PD-L1 blockade. Cancer Res.

[12] Schaue D, Ratikan JA, Iwamoto KS, McBride WH (2012). Maximizing tumor immunity with fractionated radiation. Int J Radiat Oncol Biol Phys.

[13] Vanpouille-Box C, Alard A, Aryankalayil MJ, Sarfraz Y, Diamond JM, Schneider RJ (2017). DNA exonuclease Trex1 regulates radiotherapy-induced tumour immunogenicity. Nat Commun.

[14] Kachikwu EL, Iwamoto KS, Liao Y-P, DeMarco JJ, Agazaryan N, Economou JS (2011). Radiation enhances regulatory T cell representation. Int J Radiat Oncol Biol Phys.

[15] Lan J, Li R, Yin L-M, Deng L, Gui J, Chen B-Q (2018). Targeting myeloid-derived suppressor cells and programmed death ligand 1 confers therapeutic advantage of ablative hypofractionated radiation therapy compared with conventional fractionated radiation therapy. Int J Radiat Oncol Biol Phys.

[16] Chiang C-S, Fu SY, Wang S-C, Yu C-F, Chen F-H, Lin C-M (2012). Irradiation promotes an m2 macrophage phenotype in tumor hypoxia. Front Oncol.

[17] Mirjolet C, Charon-Barra C, Ladoire S, Arbez-Gindre F, Bertaut A, Ghiringhelli F (2018). Tumor lymphocyte immune response to preoperative radiotherapy in locally advanced rectal cancer: the LYMPHOREC study. Oncoimmunology..

[18] Brenner DJ (2008). The linear-quadratic model is an appropriate methodology for determining isoeffective doses at large doses per fraction. Semin Radiat Oncol.

[19] Wong J, Armour E, Kazanzides P, Iordachita I, Tryggestad E, Deng H (2008). A high resolution small animal radiation research platform (SARRP) with x-ray tomographic guidance capabilities. Int J Radiat Oncol Biol Phys.

[20] Bray NL, Pimentel H, Melsted P, Pachter L (2016). Near-optimal probabilistic RNA-seq quantification. Nat Biotechnol.

[21] Love MI, Huber W, Anders S (2014). Moderated estimation of fold change and dispersion for RNA-seq data with DESeq2. Genome Biol.

[22] Bindea G, Mlecnik B, Hackl H, Charoentong P, Tosolini M, Kirilovsky A (2009). ClueGO: a Cytoscape plug-in to decipher functionally grouped gene ontology and pathway annotation networks. Bioinforma Oxf Engl.

[23] Dewan MZ, Galloway AE, Kawashima N, Dewyngaert JK, Babb JS, Formenti SC (2009). Fractionated but not single-dose radiotherapy induces an immune-mediated abscopal effect when combined with anti-CTLA-4 antibody. Clin Cancer Res Off J Am Assoc Cancer Res..

[24] Hiniker SM, Chen DS, Knox SJ (2012). Abscopal effect in a patient with melanoma. N Engl J Med.

[25] Golden EB, Demaria S, Schiff PB, Chachoua A, Formenti SC (2013). An abscopal response to radiation and ipilimumab in a patient with metastatic non-small cell lung cancer. Cancer Immunol Res..

[26] Frey B, Rückert M, Weber J, Mayr X, Derer A, Lotter M (2017). Hypofractionated irradiation has immune stimulatory potential and induces a timely restricted infiltration of immune cells in colon cancer tumors. Front Immunol.

[27] Ho WS, Wang H, Maggio D, Kovach JS, Zhang Q, Song Q (2018). Pharmacologic inhibition of protein phosphatase-2A achieves durable immune-mediated antitumor activity when combined with PD-1 blockade. Nat Commun.

[28] Filatenkov A, Baker J, Mueller AMS, Kenkel J, Ahn G-O, Dutt S (2015). Ablative tumor radiation can change the tumor immune cell microenvironment to induce durable complete remissions. Clin Cancer Res.

[29] Lee Y, Auh SL, Wang Y, Burnette B, Wang Y, Meng Y (2009). Therapeutic effects of ablative radiation on local tumor require CD8+ T cells: changing strategies for cancer treatment. Blood..

[30] Mantovani A, Schioppa T, Porta C, Allavena P, Sica A (2006). Role of tumor-associated macrophages in tumor progression and invasion. Cancer Metastasis Rev.

[31] Klug F, Prakash H, Huber PE, Seibel T, Bender N, Halama N (2013). Low-dose irradiation programs macrophage differentiation to an iNOS^+^/M1 phenotype that orchestrates effective T cell immunotherapy. Cancer Cell.

[32] Prakash H, Klug F, Nadella V, Mazumdar V, Schmitz-Winnenthal H, Umansky L (2016). Low doses of gamma irradiation potentially modifies immunosuppressive tumor microenvironment by retuning tumor-associated macrophages: lesson from insulinoma. Carcinogenesis..

[33] Kuang D-M, Zhao Q, Peng C, Xu J, Zhang J-P, Wu C (2009). Activated monocytes in peritumoral stroma of hepatocellular carcinoma foster immune privilege and disease progression through PD-L1. J Exp Med.

[34] Taube JM, Galon J, Sholl LM, Rodig SJ, Cottrell TR, Giraldo NA (2018). Implications of the tumor immune microenvironment for staging and therapeutics. Mod Pathol Off J U S Can Acad Pathol Inc.

[35] Cassetta Luca, Pollard Jeffrey W. (2018). Targeting macrophages: therapeutic approaches in cancer. Nature Reviews Drug Discovery.

[36] Ben-Meir K, Twaik N, Baniyash M (2018). Plasticity and biological diversity of myeloid derived suppressor cells. Curr Opin Immunol.

[37] Limagne E, Richard C, Thibaudin M, Fumet J-D, Truntzer C, Lagrange A (2019). Tim-3/galectin-9 pathway and mMDSC control primary and secondary resistances to PD-1 blockade in lung cancer patients. Oncoimmunology..

[38] Viitala MK, Virtakoivu R, Tadayon S, Rannikko J, Jalkanen S, Hollmén M. Immunotherapeutic blockade of macrophage clever-1 reactivates the CD8+ T cell response against immunosuppressive tumors. Clin Cancer Res Off J Am Assoc Cancer Res. 2019. 10.1158/1078-0432.CCR-18-3016 30755440

[39] Poleszczuk J, Enderling H (2018). The optimal radiation dose to induce robust systemic anti-tumor immunity. Int J Mol Sci.

[40] Boustani, Grapin, Laurent, Apetoh, Mirjolet (2019). The 6th R of Radiobiology: Reactivation of Anti-Tumor Immune Response. Cancers.

[41] Habets THPM, Oth T, Houben AW, Huijskens MJAJ, Senden-Gijsbers BLMG, Schnijderberg MCA (2016). Fractionated radiotherapy with 3 x 8 Gy induces systemic anti-tumour responses and abscopal tumour inhibition without modulating the humoral anti-tumour response. PLoS One.

[42] Muraro E, Furlan C, Avanzo M, Martorelli D, Comaro E, Rizzo A (2017). Local high-dose radiotherapy induces systemic Immunomodulating effects of potential therapeutic relevance in oligometastatic breast cancer. Front Immunol.

[43] Fadul CE, Fisher JL, Gui J, Hampton TH, Côté AL, Ernstoff MS (2011). Immune modulation effects of concomitant temozolomide and radiation therapy on peripheral blood mononuclear cells in patients with glioblastoma multiforme. Neuro-Oncol..

[44] Chandra RA, Wilhite TJ, Balboni TA, Alexander BM, Spektor A, Ott PA (2015). A systematic evaluation of abscopal responses following radiotherapy in patients with metastatic melanoma treated with ipilimumab. OncoImmunology..

[45] Antonia SJ, Villegas A, Daniel D, Vicente D, Murakami S, Hui R (2017). Durvalumab after Chemoradiotherapy in stage III non-small-cell lung cancer. N Engl J Med.

[46] Robert C, Long GV, Brady B, Dutriaux C, Maio M, Mortier L (2015). Nivolumab in previously untreated melanoma without BRAF mutation. N Engl J Med.

[47] Borghaei H, Paz-Ares L, Horn L, Spigel DR, Steins M, Ready NE (2015). Nivolumab versus docetaxel in advanced nonsquamous non–small-cell lung cancer. N Engl J Med.

[48] Yu X, Harden K, Gonzalez LC, Francesco M, Chiang E, Irving B (2009). The surface protein TIGIT suppresses T cell activation by promoting the generation of mature immunoregulatory dendritic cells. Nat Immunol.

[49] Josefsson SE, Beiske K, Blaker YN, Førsund MS, Holte H, Østenstad B (2019). TIGIT and PD-1 mark intratumoral T cells with reduced effector function in B-cell non-Hodgkin lymphoma. Cancer Immunol Res..

[50] Casado JG, Pawelec G, Morgado S, Sanchez-Correa B, Delgado E, Gayoso I (2009). Expression of adhesion molecules and ligands for activating and costimulatory receptors involved in cell-mediated cytotoxicity in a large panel of human melanoma cell lines. Cancer Immunol Immunother CII.

[51] Stanietsky N, Simic H, Arapovic J, Toporik A, Levy O, Novik A (2009). The interaction of TIGIT with PVR and PVRL2 inhibits human NK cell cytotoxicity. Proc Natl Acad Sci U S A.

[52] Kong Y, Zhu L, Schell TD, Zhang J, Claxton DF, Ehmann WC (2016). T-cell immunoglobulin and ITIM domain (TIGIT) associates with CD8+ T-cell exhaustion and poor clinical outcome in AML patients. Clin Cancer Res Off J Am Assoc Cancer Res.

[53] Johnston RJ, Comps-Agrar L, Hackney J, Yu X, Huseni M, Yang Y (2014). The Immunoreceptor TIGIT regulates antitumor and antiviral CD8 + T cell effector function. Cancer Cell.

