# Supplementary material for: Optimized fractionated radiotherapy with anti-PD-L1 and anti-TIGIT: a promising new combination
Source: J Immunother Cancer. 2019 Jun 25;7:160. doi: 10.1186/s40425-019-0634-9 (PMC6593525; doi:10.1186/s40425-019-0634-9)
Supplement: Supplementary file 2 — Figure S1. Gating strategy for lymphoid cells identification and quantification in tumor tissue. Figure S2. Gating strategy for myeloid and tumor cells identification, quantification and phenotype (PD-L1 expression) in tumor tissue. Figure S3. Gating strategy for lymphoid cells functionality quantification in tumor tissue. Figure S4. Efficacy evaluation of immunotherapy (anti-PD-L1 and/or anti-TIGIT) and RT (3x8Gy) in B16-F10 model. Growth of irradiated tumors in mice treated with IgG + 0Gy (black), IgG + 3x8Gy (red), anti-TIGIT + anti-PD-L1 (blue) and with anti-TIGIT + anti-PD-L1 + 3x8Gy (purple). Mean ± SEM. X axes express the number of days since the beginning of RT. Y axes express the tumor volume (mm3). Experimental groups contained at 5 mice per group. Non-parametric Mann-Whitney test was used. Figure S5. Immunomonitoring of Treg and CD8+ T cells and their KI67 and PD-1 status after radiotherapy. Ten days after the injection of CT26 colon murine cancer, mice were assigned in 4 groups: control (at day 7), 1 × 16.4Gy (red), 3x8Gy (blue), 18x2Gy (purple) (a). Seven, 14 and 30 days after the beginning of RT, flow cytometry monitoring (FCM) was performed on dissociated tumors. CD8+ T cells (a) and Treg T cells (b) were analyzed according to their status for KI67 and PD-1 labelling. All data are shown with box and whiskers with min to max values obtained from 8 independent samples per point (duplicate, n = 8 per condition). In the second part of a) and b), representative cytometry analysis was highlighted for each condition at day 7 and day 14 after treatment. *p < 0.05. Non-parametric Mann-Whitney test was used. (ZIP 3298 kb) [file 40425_2019_634_MOESM2_ESM.pptx]

## Slide 1
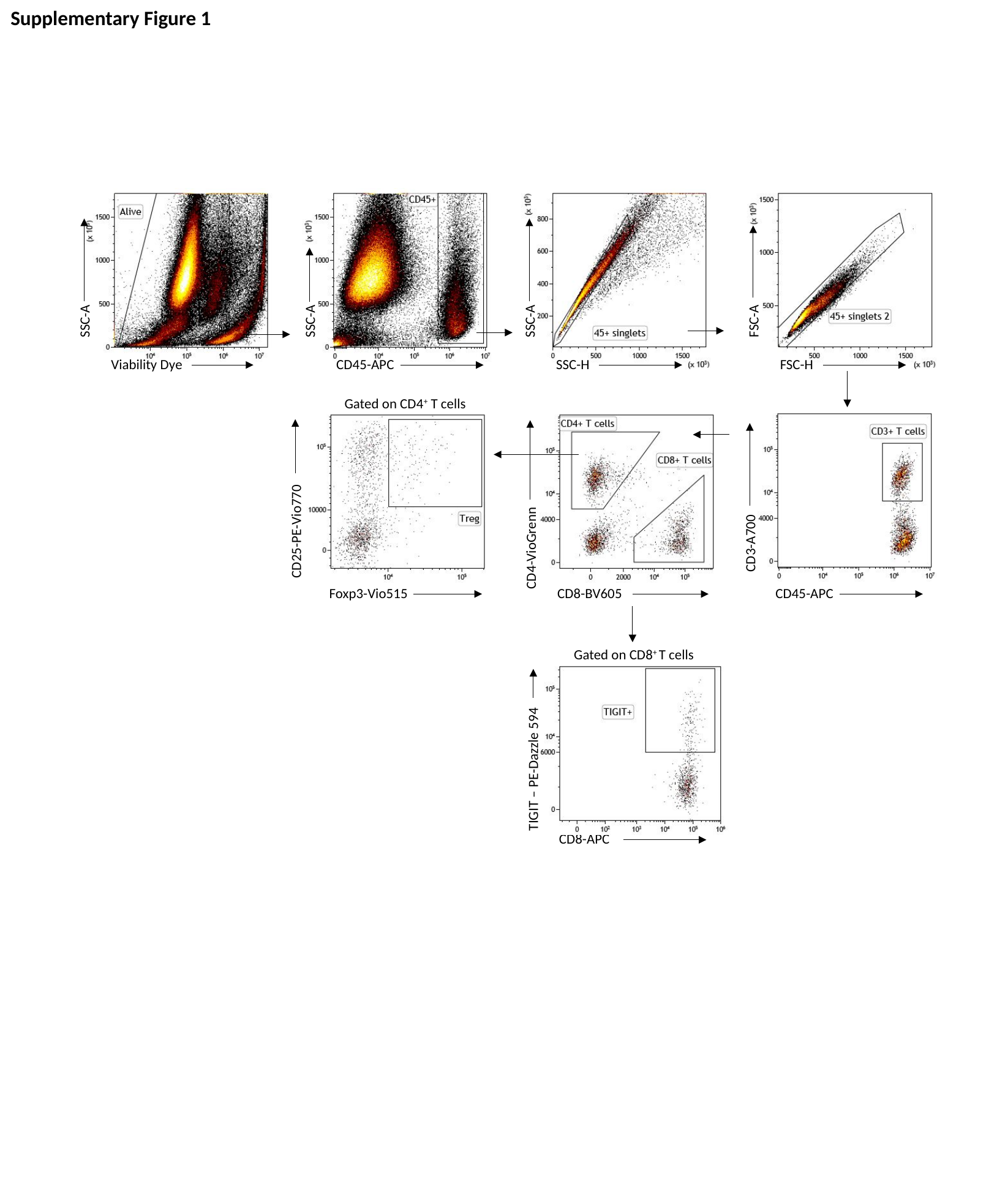

Supplementary Figure 1
SSC-A
SSC-A
SSC-A
FSC-A
Viability Dye
CD45-APC
SSC-H
FSC-H
Gated on CD4+ T cells
CD25-PE-Vio770
CD3-A700
CD4-VioGrenn
Foxp3-Vio515
CD8-BV605
CD45-APC
Gated on CD8+ T cells
TIGIT – PE-Dazzle 594
CD8-APC

## Slide 2
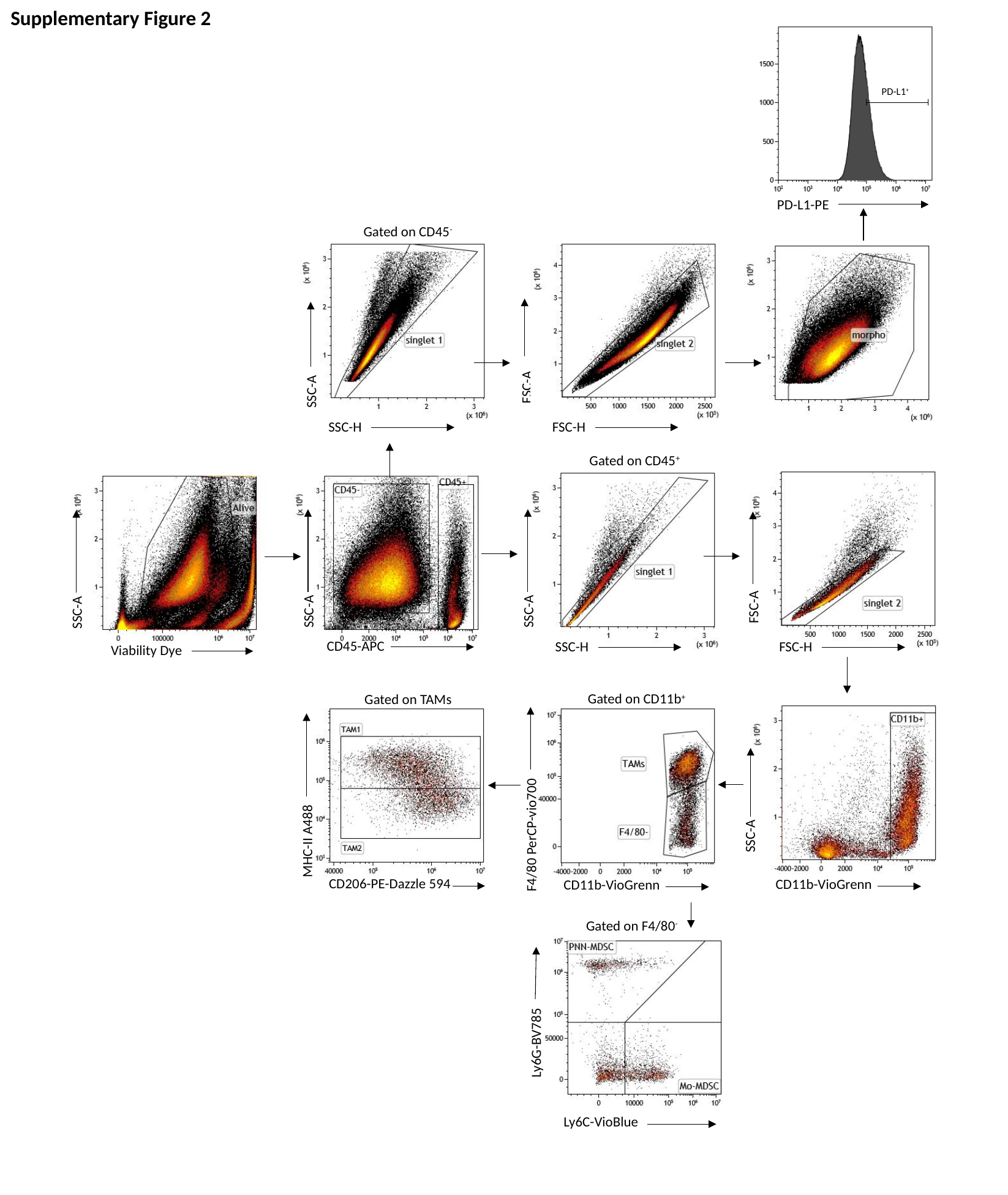

Supplementary Figure 2
PD-L1+
PD-L1-PE
Gated on CD45-
FSC-A
SSC-A
SSC-H
FSC-H
Gated on CD45+
FSC-A
SSC-A
SSC-A
SSC-A
CD45-APC
SSC-H
FSC-H
Viability Dye
Gated on CD11b+
Gated on TAMs
F4/80 PerCP-vio700
SSC-A
MHC-II A488
CD206-PE-Dazzle 594
CD11b-VioGrenn
CD11b-VioGrenn
Gated on F4/80-
Ly6G-BV785
Ly6C-VioBlue

## Slide 3
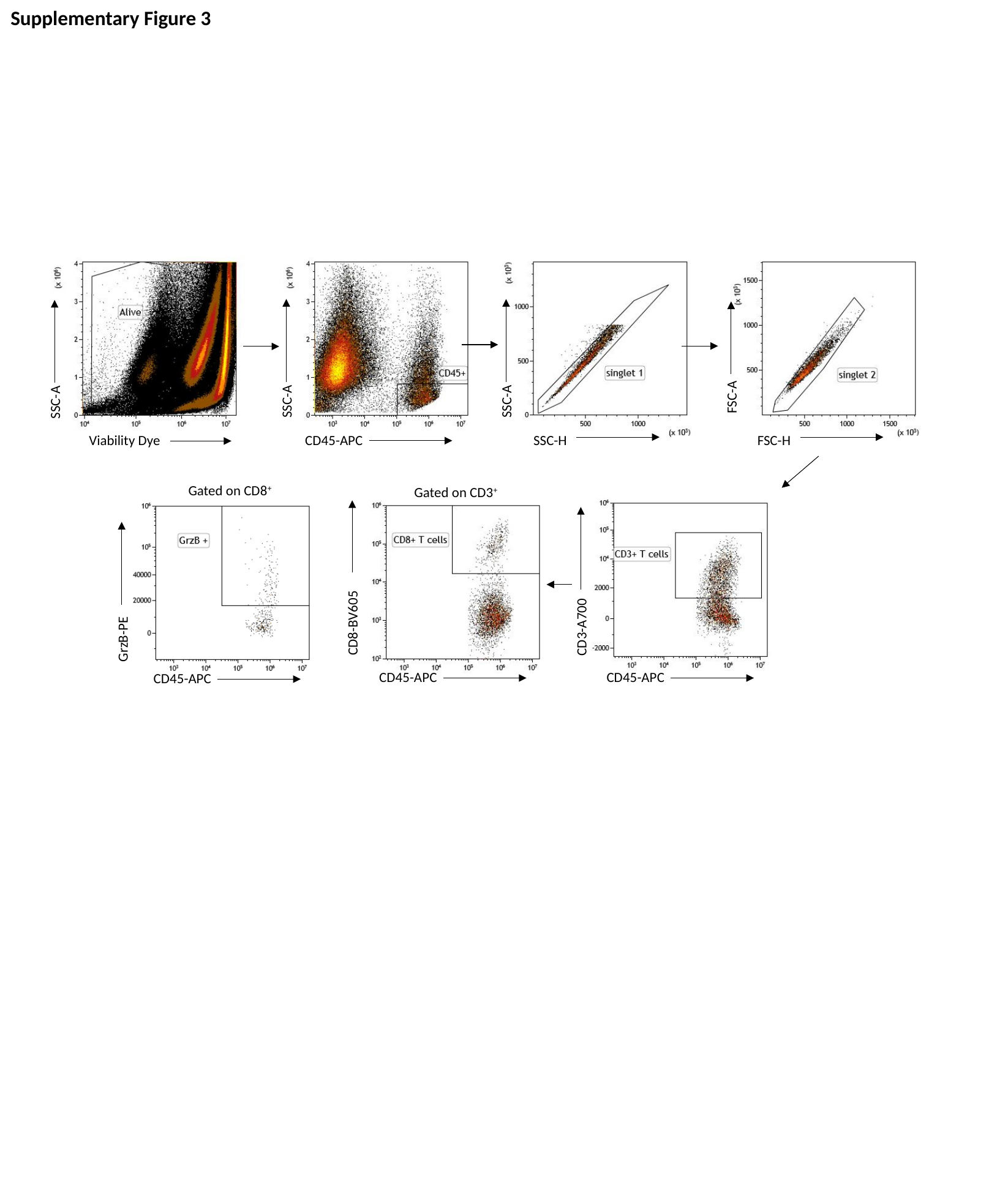

Supplementary Figure 3
FSC-A
SSC-A
SSC-A
SSC-A
Viability Dye
CD45-APC
SSC-H
FSC-H
Gated on CD8+
Gated on CD3+
CD8-BV605
CD3-A700
GrzB-PE
CD45-APC
CD45-APC
CD45-APC

## Slide 4
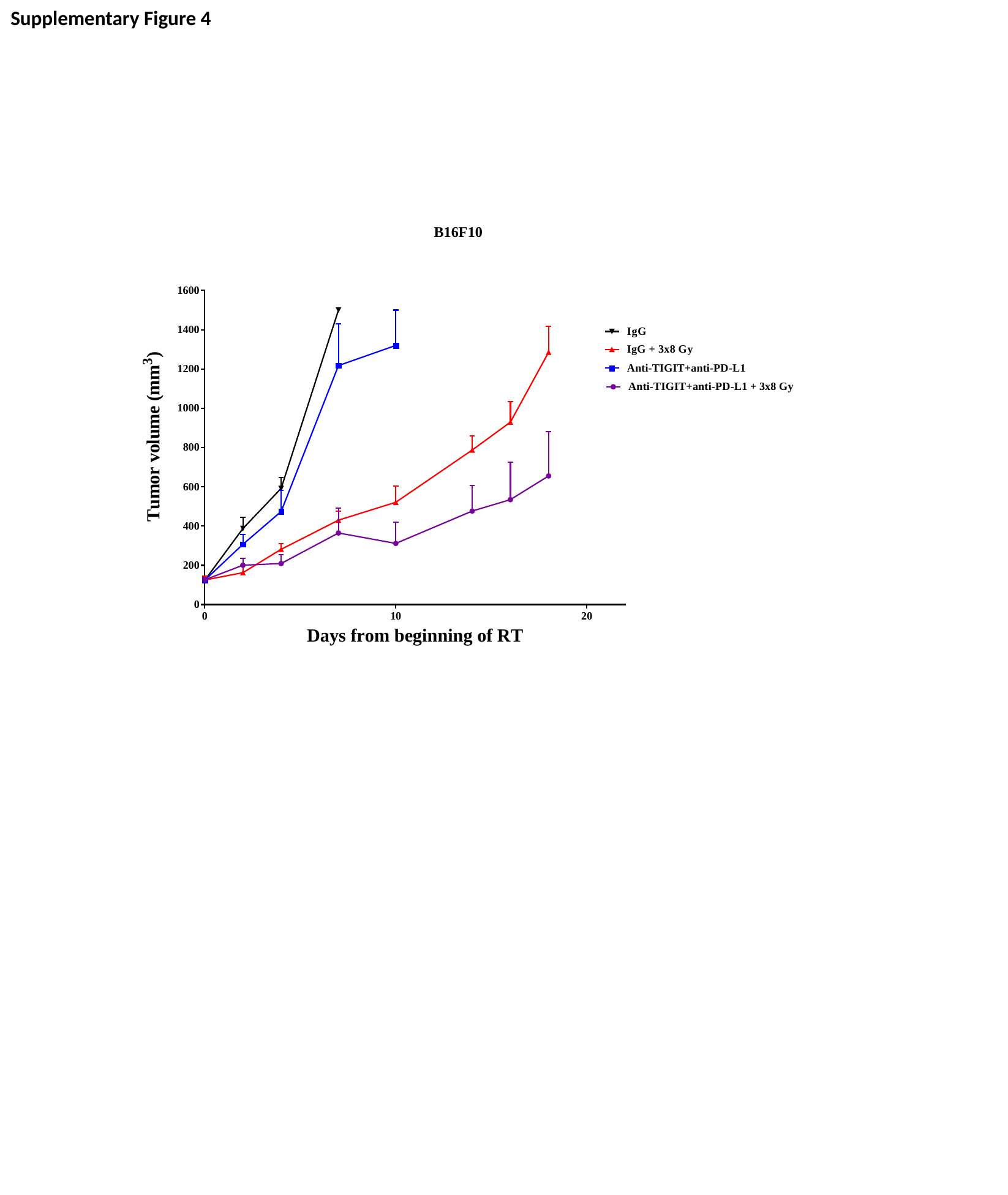

Supplementary Figure 4
